# Impact of breast magnetic resonance imaging on the locoregional staging and management of breast cancer

**DOI:** 10.1590/0100-3984.2018.0064

**Published:** 2019

**Authors:** Luciana Karla Lira França, Almir Galvão Vieira Bitencourt, Fabiana Baroni Alves Makdissi, Carla Curi, Juliana Alves de Souza, Elvira Ferreira Marques

**Affiliations:** 1 Hospital Memorial Arthur Ramos, Maceió, AL, Brazil.; 2 A.C.Camargo Cancer Center, São Paulo, SP, Brazil.

**Keywords:** Breast neoplasms, Magnetic resonance imaging, Neoplasm staging, Breast/diagnostic imaging, Neoplasias de mama, Ressonância magnética, Estadiamento de neoplasias, Mama/diagnóstico por imagem

## Abstract

**Objective:**

To assess the impact of magnetic resonance imaging (MRI) on the locoregional staging of breast cancer.

**Materials and Methods:**

We evaluated 61 patients with breast cancer who underwent pre-treatment breast MRI, between August 2015 and April 2016. An experienced breast surgeon determined the surgical treatment, on the basis of the findings of conventional imaging examinations, and made a subsequent treatment recommendation based on the MRI findings, then determining whether the MRI changed the approach, as well as whether it had a positive or negative impact on the treatment.

**Results:**

The mean age was 50.8 years (standard deviation, 12.0 years). The most common histological type was invasive breast carcinoma of no special type (in 68.9%), and the most common molecular subtype was luminal B (in 45.9%). Breast MRI modified the therapeutic management in 23.0% of the cases evaluated, having a positive impact in 82.7%.

**Conclusion:**

Breast MRI is an useful tool for the locoregional staging of breast cancer, because it provides useful information that can have a positive impact on patient treatment.

## Abstract (pt)

**Objetivo:**

Avaliar o impacto do uso da ressonância magnética (RM) no estadiamento pré-operatório do câncer de mama.

**Materiais e Métodos:**

Foram avaliadas 61 pacientes com carcinoma mamário submetidas a RM das mamas, no período de agosto de 2015 a abril de 2016. Um mastologista foi questionado sobre a conduta terapêutica indicada diante dos dados da paciente e dos exames convencionais e, após realização da RM, foi novamente questionado sobre a conduta para determinar se houve mudança e impacto no tratamento.

**Resultados:**

A média de idade das pacientes foi 50,8 anos (desvio-padrão: 12,0), o tipo histológico mais frequente foi carcinoma mamário invasivo tipo não especial (68,9%) e o imunofenótipo mais prevalente foi luminal B (45,9%). A RM das mamas mudou a conduta terapêutica em 23% dos casos, com impacto positivo em 82,7%.

**Conclusão:**

A RM das mamas é instrumento útil no estadiamento locorregional do câncer de mama, sendo capaz de adicionar informações que tenham impacto positivo no tratamento.

## INTRODUCTION

Breast cancer is the leading cause of cancer death in women in Brazil. In 2013, it achieved a world population age-adjusted mortality rate of 12.66/100,000 women, which justifies the growing efforts to improve diagnostic methods and treatment^(^^1^^)^. The basic diagnostic assessment for breast cancer includes clinical examination, mammography, and ultrasound to define the extent of the disease^(^^2^^)^.

Breast-conserving surgery is currently the management practice of choice for early-stage breast cancer, because, when combined with radiation therapy, it has survival rates similar to those of mastectomy, provided there is appropriate patient selection. In this scenario, magnetic resonance imaging (MRI) of the breast gains prominence because it has over 90% sensitivity and is superior to other conventional imaging methods in determining tumor size and identifying additional lesions^(^^3^^,^^4^^)^. MRI identifies additional ipsilateral foci in 12.0-31.2% of breast cancer patients and contralateral foci in 3-10%, leading to changes in treatment in up to one third of them^(^^5^^-^^7^^)^.

It is worth noting that breast cancer is a heterogeneous clinical entity that should not be assessed as a single disease. Different histological and molecular subtypes of breast cancer have different imaging findings, prognoses, and therapeutic responses. Therefore, it would be helpful if studies evaluating breast MRI were individualized for each subtype, so as to define in which scenarios this imaging method performs best. For example, invasive lobular carcinoma (ILC) is a histological type of breast cancer that presents higher rates of multicentricity and bilateralism, the extent of the disease therefore being more likely to be underestimated by conventional imaging methods, so the use of preoperative MRI for the staging of ILC is recommended^(^^8^^)^. In addition to the histological type, the molecular subtype has been increasingly taken into account to define the management of breast cancer. The molecular subtype determines the biological behavior of the disease, be it in imaging tests, probability of multicentricity/bilaterality, or prognostics, which indicates the need for subgroup-specific research^(^^9^^)^.

The objective of the present study was to evaluate the impact of MRI on the therapeutic management of breast cancer. We also attempted to determine whether a change in therapeutic management correlates with the histological type or molecular subtype of the tumors.

## MATERIALS AND METHODS

This was a prospective, single-center, cross-sectional study involving patients diagnosed with breast carcinoma who underwent breast MRI for the locoregional staging of the disease between August 2015 and April 2016. During that period, we evaluated 69 patients with breast neoplasms submitted to pretreatment MRI of the breast. Of those, we excluded six for having received part of their treatment at another center, one for not having test data in her medical record, and one for not having lesions that were detectable on conventional imaging methods. Therefore, the final sample comprised 61 patients. For all of those patients, we assessed demographic profiles, personal/family history of cancer, physical examination findings, imaging findings, histopathological findings, and immunohistochemical data.

To acquire the MRI sequences, we used a 1.5 T scanner (Signa HDxt; GE Healthcare, Milwaukee, WI, USA) with a dedicated coil. Unenhanced axial three-dimensional (3D) T1-weighted gradient-echo sequences with a slice thickness of 2.5 mm and sagittal T2-weighted short-tau inversion-recovery sequences with a slice thickness of 4 mm were acquired for both breasts. For the dynamic study, five axial fat-suppressed 3D T1-weighted gradient-echo sequences were obtained. The first sequence was acquired prior to contrast injection, the second was acquired 20 s after injection of the paramagnetic contrast agent gadopentetate dimeglumine (20 mL at a rate of 3 mL/s), the remaining sequences being sequentially acquired in the following minutes. From these dynamic images, post-processing images were generated by subtracting the unenhanced sequence from the contrast-enhanced sequences, in order to improve visualization of the enhanced areas. The last sequence acquired comprised contrast-enhanced sagittal fat-sat 3D T1-weighted gradient-echo images of both breasts, with a slice thickness of 1 mm.

The histopathological diagnosis of breast cancer was confirmed by percutaneous biopsy performed before treatment initiation. Information on the histological and molecular subtypes was obtained from the reports issued by the pathology department of our center.

Two radiologists with experience in breast imaging evaluated the MRI sequences in order to characterize the main lesion and to investigate additional lesions, following the Breast Imaging Reporting and Data System (BI-RADS) criteria^(^^10^^)^. We asked a breast surgeon, who was blinded to the MRI data, to recommend a treatment regimen based on a review of the clinical data, mammography findings, ultrasound findings, and results of the pathology study of the biopsy sample. We then revealed the MRI data and asked the breast surgeon what would be the final proposed management strategy, based on institutional protocols. Subsequently, the practices proposed before and after review of the MRI data were compared to determine if there had been any change. Two evaluators reviewed the cases to determine if the MRI findings had had any impact, and divided them into the following categories:

- No impact: MRI findings matched those of the physical examination, mammography, and ultrasound, or they did not modify the proposed treatment.
- Positive impact: MRI detected additional lesions or a larger tumor size, leading to changes in management that were later proven appropriate by the final pathology study.
- Negative impact: MRI findings led to more extensive surgery, but the findings were later proven to be false-negatives or the final pathology study showed the lesions to be smaller than expected.

The collected data were entered into the SPSS Statistics software package, version 20.0 (IBM Corp., Armonk, NY, USA) for statistical analysis. A descriptive analysis of the categorical variables was conducted on the basis of the calculation of absolute and relative frequencies. Numerical variables were described as means, standard deviations, minimums, and maximums. To determine the size of the main tumor, the longest axis as measured by the MRI scan and by the other conventional tests was compared with the largest measurement of the surgical specimen described in the pathology report. A multivariate analysis was conducted to investigate factors related to the changes in management. A probability of type I error below or equal to 5% was considered statistically significant (*p* ≤ 0.05).

## RESULTS

The mean age of the patients was 50.8 ± 12.0 years (range, 30-74 years). Of the 61 patients evaluated, 27 (44.3%) were < 50 years of age at diagnosis. The most common histological subtype was invasive breast carcinoma of no special type, which was seen in 42 (68.9%) of the patients, followed by ILC in 6 (9.8%), ductal carcinoma in situ (DCIS) in 4 (6.6%), and other special types in 9 (14.8%). Among the invasive carcinomas, the most common molecular subtype was luminal B, which was seen in 28 (45.9%) of the patients, followed by luminal A in 12 (19.7%), triple-negative in 10 (16.4%), and HER-2 in 7 (11.5%).

On MRI, the index lesion appeared as a nodule in 50 patients (82%), non-nodular enhancement in 10 (16.4%), and negative in 1 (1.6%), whereas (in all cases) it was identified as DCIS on mammography (calcifications) and in the pathology study. The mean lesion size on MRI was 29.9 mm (range, 5-102 mm).

**Figure 1:**
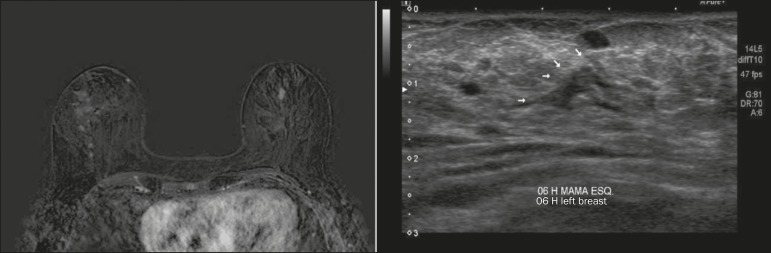
Fat-saturated T1-weighted sequences with digital subtraction, showing non-nodular enhancement in the right breast diagnosed as invasive lobular carcinoma, and, in the left breast, an irregular nodule, which was not detected on conventional imaging tests but which was seen as an irregular intraductal nodule on second-look ultrasound, consistent with the MRI findings. Histological diagnosis of DCIS.

MRI identified additional ipsilateral lesions in 13 (21.3%) of the 61 cases and unusual lymph nodes not previously characterized in 5 (8.2%). It also showed suspicious findings in the contralateral breast in 5 cases (8.2%). Of the total number of additional lesions identified on MRI and submitted to pathology studies before initiation of treatment (seven lesions), two (28.5%) were confirmed as neoplasms-one DCIS and one axillary lymph node with positive cytology for malignancy. Of the five patients with suspicious (BI-RADS 4) contralateral findings, four (80%) were submitted to a second-look ultrasound and only one did not present a corresponding finding (Table 1). In one (20%) of the suspicious contralateral lesions identified, the histological diagnosis was DCIS (Figure 1). The patient in whom the second-look ultrasound results were negative received treatment for the index breast tumor and, at this writing, has been under follow-up for two years, during which time the contralateral lesion disappeared and has not come back. The patient in whom the contralateral lesion was not investigated underwent neoadjuvant chemotherapy (NAC), and, although she had a partial response in the index breast, the contralateral finding has not been seen in the follow-up tests for two years.

**Table 1:** Second-look ultrasound of additional suspicious contralateral lesions.

	MRI finding	Second look ultrasound	Pathology result
1	Non-nodular enhancement	Not performed	Study not performed;
			Disappearance after NAC;
			Negative tests during two years of follow-up
2	Irregular nodule	Irregular nodule	Usual ductal hyperplasia
3	Irregular nodule	Irregular intra-ductal nodule	DCIS
4	Non-nodular enhancement	Negative	Study not performed;
			Disappearance after NAC;
			Negative tests during two years of follow-up
5	Non-nodular enhancement	Ill-defined area	Stromal fibrosis

MRI led to changes in management in 14 patients (23%): 5 (8.2%) were referred for mastectomy; 5 (8.2%) were referred for NAC; 1 (1.6%) was referred for more extensive breast-conserving surgery; 1 (1.6%) was referred for contralateral surgery; 1 (1.6%) was referred for axillary lymph node dissection; and 1 (1.6%) was referred for less extensive breast-conserving surgery. In the last case, MRI led to less extensive breast-conserving surgery because the patient presented a nodule < 1.0 cm that was diagnosed as malignant and an area of BI-RADS 4 microcalcifications in the same breast that were diagnosed as benign after a histological study. That finding generated anxiety due to its extent and the risk of underestimation. However, no abnormal enhancement was seen in the area and it was possible to perform surgery aimed at nodule resection with tumor-free margins.

In the 14 cases in which MRI led to changes in the therapeutic management, it had a positive impact in 12 (85.7%) and a negative impact in 2 (14.3%), as shown in Table 2. Those two patients had MRI findings suggestive of multicentricity and were therefore submitted to mastectomy. However, multicentricity was not confirmed in the surgical specimen.

**Table 2:** Cases in which MRI led to change in management and the corresponding impact on patient treatment.

	Initial therapeutic proposal	MRI finding that led to change in management	Post-MRI therapeutic proposal	Impact
1	Breast-conserving surgery	Multicentric disease	Mastectomy	Negative: no multicentricity identified in the specimen
2	Mastectomy	Suspicious axillary lymph node	Mastectomy + axillary lymph node dissection	Positive: reduces surgical time and allows for better preoperative planning
3	Breast-conserving surgery	Multicentric disease	Mastectomy	Negative: no multicentricity identified in the specimen
4	Breast-conserving surgery	Microcalcifications with negative biopsy, no enhancement on MRI	Less-extensive breast-conserving surgery	Positive
5	Breast-conserving surgery	Larger tumor size	More-extensive breast-conserving surgery	Positive: pathology study of the specimen consistent with MRI measurements
6	Breast-conserving surgery	Contralateral tumor	Contralateral surgery	Positive: tumor-free resection margins of DCIS in the contralateral breast
7	Breast-conserving surgery	Larger tumor size	Mastectomy	Positive: pathology study of the specimen consistent with MRI measurements
8	Breast-conserving surgery	Larger tumor size	NAC	Positive
9	Breast-conserving surgery	Multicentric disease	Mastectomy	Positive: pathology study of the surgical specimen consistent with MRI measurements
10	Breast-conserving surgery	Multifocal disease	NAC	Positive
11	Breast-conserving surgery	Suspicious axillary lymph node	NAC	Positive
12	Breast-conserving surgery	Suspicious axillary lymph node	NAC	Positive
13	Breast-conserving surgery	Changed axillary staging N1→N2	NAC	Positive
14	Breast-conserving surgery	Multifocal disease	Mastectomy	Positive: pathology study of the surgical specimen consistent with MRI measurements

With regards to the histological type, MRI led to changes in management in 50% of the patients with ILC (contralateral surgery added in 33% and breast-conserving surgery switched to mastectomy in 66%); in 19% of the patients with invasive carcinoma of no special type (breast-conserving surgery switched to mastectomy in 37.5% and to NAC in 62.5%); and in 25% of the patients with DCIS (all switched to more extensive breast-conserving surgery), although that was not considered statistically significant (*p* = 0.414). As for the molecular subtypes of invasive carcinomas, those with the highest rates of change in management were luminal B (accounting for 30% of the cases in which MRI led to a change in management-75% of them switched from breast-conserving surgery to NAC and 25% from breast-conserving surgery to mastectomy), followed by HER-2 tumors (accounting for 20% of the cases in which there was change in management after MRI-all cases switched from breast-conserving surgery to mastectomy; *p* = 0.283).

## DISCUSSION

Breast MRI is an important tool for the locoregional staging of breast neoplasms, because, in addition to being able to provide information on tumor morphology and size, it has high sensitivity and can detect additional lesions that are otherwise occult^(^^4^^,^^11^^-^^16^^)^. In the present study, additional suspicious lesions in the ipsilateral breast were identified in 21.3% of the cases and, in the contralateral breasts, in 8.2% of them, rates similar to those found in the literature. In a meta-analysis evaluating 40 studies on MRI detection of additional ipsilateral lesions in patients with breast neoplasms, Piana et al. found a mean detection rate of 20% (range, 6-70%), with a positive predictive value of 67%. An analysis of 30 studies on the identification of additional contralateral lesions found a mean detection rate of 5.5% (range, 2.3-22%), with a positive predictive value of 37%^(^^17^^)^.

Although breast MRI has high sensitivity, it has relatively low specificity and not enough accuracy for its findings to be considered diagnostic^(^^18^^)^; histological confirmation is therefore necessary to define the most appropriate management^(^^19^^)^. Because MRI-guided biopsies are costly and are not widely available, a viable solution is to use targeted ultrasound to characterize the lesions and then perform a biopsy. When investigating MRI-detected lesions with second-look ultrasound, Aracava et al.^(^^20^^)^ demonstrated that 100% of BI-RADS 5 findings and 90% of BI-RADS 4 findings were seen on the second-look ultrasound, indicating that this type of test has a high probability of identifying suspicious lesions.

In the present study, four (80%) of the five suspicious contralateral findings were submitted to second-look ultrasound. Although our sample was small, three (75%) of the suspicious MRI-detected lesions were identified on ultrasound, one of those three (33.3%) testing positive for malignancy in the pathology study. Our findings are comparable to those of Hong et al.^(^^21^^)^, who used second-look ultrasound to assess 121 MRI-detected suspicious lesions and found corresponding lesions on ultrasound in 105 (86.8%), of which 29 (27.9%) were proven to be malignant.

In our sample, MRI led to a change in management in 23% of the patients submitted to breast MRI and, in 35.7%, that change was a switch from breast-conserving surgery to mastectomy. In a retrospective study involving 160 patients, França et al.^(^^4^^)^ identified MRI as a decisive factor for a change in the therapeutic management in 14.4% of cases, lower than the rate we found in our study. However, that was a retrospective study with no mention of standardization of management by the same evaluator or of the axillary status. Gonzalez et al.^(^^22^^)^ evaluated 220 patients submitted to breast MRI before the initiation of treatment for breast cancer and observed a change in the proposed management in 40 (18%). The rate of change in management in the study conducted by Mukherjee et. al.^(^^23^^)^-66% for the first evaluator and 41% for the second-was higher than that found in our sample, and the most common change was a switch from breast-conserving surgery to mastectomy.

Piana et al.^(^^17^^)^ conducted a systematic review of the literature regarding the impact of MRI on the planning of breast cancer surgery and found that the conversion to mastectomy was appropriate, as defined by the subsequent histopathological study, in 8.3% of the cases and inappropriate in 1.7%. In our study, the conversion to mastectomy was deemed appropriate in three (4.9%) of the 61 cases and inappropriate in two (3.3%). In both of the cases in which mastectomy was considered inappropriate, MRI suggested multicentric disease, which was not investigated preoperatively. Therefore, given its current sensitivity and specificity, MRI cannot replace pathology in the diagnostic confirmation of additional lesions. All additional lesions detected on MRI should, therefore, be confirmed by biopsy in order to spare patients more aggressive surgeries that are unnecessary. However, our study did not analyze factors such as family risk, patient choice, or other patient-related conditions that may have justified the change in management in these specific cases.

The use of MRI in the diagnostic assessment led to a less extensive breast-conserving surgery in one patient (1.6%) who had a large ipsilateral cluster of microcalcifications that, despite the benign results of the preoperative biopsy, generated anxiety due to its distribution and extent. Before MRI, the breast surgeon consulted considered performing a surgical procedure that would encompass the area of microcalcifications. However, after MRI showed no abnormal enhancement in the area, the surgeon directed the surgical procedure only for the lesion with established diagnosis.

In our study sample, a change in therapeutic management did not correlate significantly with the histological types or molecular subtype of the tumor. The ILC patients had a higher rate of change in management (50%), although the difference was not statistically significant, probably because of the small sample size.

The results of this study should be considered in the context of its limitations. The growing use of NAC in clinical practice was noted in the studied sample (34.4%), which made it impossible to confirm the multifocality/multicentricity of the cases that were not investigated prior to the institution of therapy. Our study also has other limitations: the small number of patients in the study, which makes it difficult to obtain statistically significant values; the lack of long-term follow-up, which prevents us from evaluating outcomes such as recurrence and survival; and the lack of further investigation of some MRI-detected lesions, given that the evaluator needs that information to define the most appropriate management.

There is still considerable controversy in the literature related to the routine use of breast MRI for the staging and therapeutic planning of breast cancer. Although MRI is more accurate than are other conventional methods in evaluating the extent of the disease and various retrospective studies have shown its benefits, the first prospective studies (the MONET and COMICE trials) showed no benefits in terms of reducing the rates of reoperation, positive margins, local recurrence, and mortality^(^^24^^-^^27^^)^. That is mainly due to methodological problems in those studies, namely the inclusion of centers with limited experience in the use of breast MRI and the lack of preoperative investigation of MRI-detected lesions. In addition, many factors related to treatment and to the surgical technique employed can influence the results. For example, McCahill et al.^(^^28^^)^ showed there is a substantial variation in reoperation rates after breast-conserving surgeries between surgeons and among institutions, which could hinder the analysis of the impact of MRI on this type of outcome.

Our study shows that MRI plays an important role in the preoperative staging of breast cancer, modifying the therapeutic planning in approximately 25% of the cases and having a positive impact on the vast majority of them. The current trend is for MRI to be indicated for specific groups of patients who may benefit the most from it. In addition, some considerations should be kept in mind when ordering MRI for therapeutic planning purposes: MRI findings that will affect treatment should preferably be confirmed by histological studies prior to the therapeutic definition; a preoperative biopsy or MRI-guided localization of the lesions identified only on MRI should be performed; the investigation of MRI findings should not delay treatment; and changes in treatment should be discussed with a multidisciplinary team including radiologists, pathologists, breast surgeons, oncologists, and radiotherapists.
